# Adherence to treatment for hypothyroidism in pregnancy and relationship with thyrotropin control: a retrospective observational cohort study

**DOI:** 10.1186/s12884-022-04483-8

**Published:** 2022-03-01

**Authors:** Júlia Siscart, Míriam Orós, M. Catalina Serna, Dani Perejón, Leonardo Galván, Marta Ortega

**Affiliations:** 1 Primary Care Research Institute IDIAP Jordi Gol, Catalan Institute of Health, Lleida, Spain; 2 Eixample Health Center, Catalan Institute of Health, Lleida, Spain; 3 Family Medicine Department, University of Lleida, Lleida, Spain; 4 Departament de Salut Spain, Lleida, Spain; 5 Therapeutic Research Group in Primary Care (GRETAP), Catalan Institute of Health, Lleida, Spain

**Keywords:** Pregnancy, Hypothyroidism, Levothyroxine, Thyroid stimulating hormone, Adherence to treatment

## Abstract

**Background:**

Hypothyroidism is the second most common endocrinological disease during pregnancy, with percentages that can range between 3.2 and 5.5%. A good maternal and foetal health outcome depends on thyroid hormone replacement therapy. The goal of such therapy is to maintain thyrotropin (TSH) in a range that is specific for pregnant women and varies between the trimesters of pregnancy. In our study, we wanted to analyse the adherence to hypothyroidism treatment among pregnant women and to evaluate the degree of control of the disease.

**Methods:**

We performed a retrospective observational cohort study in pregnant women between 2012 and 2018 in the Lleida health region. Therapeutic adherence was analysed by the proportion of days covered (PDC). The relationship with other variables was assessed using the regression coefficients and their 95% confidence interval (CI).

**Results:**

We examined a sample of 17,281 women, representing more than 92% of the pregnant women in the Lleida health region in the period analysed. Among this sample, the mean prevalence of hypothyroidism was 6.52% (0.07% clinical and 6.45% subclinical). 3.3% of the 17,281 pregnant women were treated. Among them, the mean adherence score was 79.6 ± 22.2. Of these, 54% presented high adherence. The latter had a higher mean age and better TSH control, in comparison to the ones showing low adherence.

**Conclusions:**

Half of the treated patients had good adherence to treatment and a better TSH control, in comparison to the others. Most of them achieved a good control at the third trimester of pregnancy.

**Supplementary Information:**

The online version contains supplementary material available at 10.1186/s12884-022-04483-8.

## Introduction

Hypothyroidism is defined as a decrease in the function of the thyroid gland and thyroid hormone in blood (T4). During pregnancy, it is the second most prevalent disease after diabetes mellitus [1, 2], with percentages that can range between 3.2 and 5.5% in Spain, depending on the region [3, 4]. In many studies, clinical and subclinical hypothyroidism have been associated with adverse effects in pregnancy, such as miscarriage and preterm birth [5, 6]. In addition, low T4 levels in pregnant women have been correlated with the presentation of long-term neuro-cognitive problems for the newborn [7, 8]. Therefore, a good maternal and foetal health outcome depends on treating maternal hypothyroidism by administrating thyroid hormone (T4). The goal of treatment is to maintain the mother’s serum TSH in the population- and trimester-specific reference range [9].

Several studies suggest that in current practice hypothyroidism may be overdiagnosed and overtreated during pregnancy. Randomized controlled clinical trials have demonstrated that treating pregnant women with subclinical hypothyroidism does not benefit either the mother or the fetus [10–12].

The World Health Organization (WHO) defines adherence as the behaviour of a person taking medications, following a diet, and/or making changes in lifestyle, in agreement with the recommendations made by health professionals [13].

There are few published studies that analyse adherence in pregnancy. In a study by the *North Jutland Prescription* in 2001 in Denmark, an adherence of 43% was estimated for the treatment of various pathologies that are prevalent during pregnancy [14]. There are other studies, such as the one carried out in an Australian health centre in which the rate of non-adherence to treatment of chronic diseases was estimated at 59.1%. The main causes of non-adherence were forgetfulness and concerns about possible side effects [15].

Lastly, thyroid hormone requirements increase during pregnancy and hypothyroidism is a fairly common disease with serious consequences in both pregnant women and newborns. In this context, we set the objective to analyse adherence to hypothyroid treatment in pregnant women and assess the degree of hypothyroidism control.

## Methods

### Study design and data collection

We performed a retrospective observational cohort study in pregnant women between 2012 and 2018 in the Lleida health region (Spain).

The data of women who gave birth at the Arnau de Vilanova Hospital from January 1, 2012 to December 31, 2018 were obtained from several different sources: the CMBD (“Conjunt Minim de Base de Dades”) database, which included all single and multiple pregnancies, live births and miscarriages; the E-CAP computerized database of medical history from the Catalan Health Institute, collecting data of patients assigned to primary care units; and the database of the Servei Català de Salut, that collects the data of completed prescriptions from the Social Security. All patients are assigned an individual code (“Codi d’identificació personal”) which is applied in all the databases.

### Study population

As inclusion criteria, women who gave birth between January 1, 2012 and December 31, 2018 were studied. Pregnancy data from the date of the last menstrual period to the date of delivery were included; therefore, data from 2011 were reviewed for pregnant women with the delivery date in 2012 and the date of the last menstruation in 2011. Pregnant women who did not belong to the Lleida health region were excluded. To evaluate the representativeness of the sample, the percentage of births studied (births registered at the Arnau de Vilanova University Hospital in Lleida) was calculated with respect to the total births in the Lleida health region in the same period. The calculation was done according to the data obtained from the “ Instituto de Estadística de Catalunya” (Idescat) (Supplementary table 1).

### Variables measured

We recorded different variables: the presence of hypothyroidism, which corresponds to code E03.9 and E02 of the ICD-10; the levels of TSH and T4 in blood at each trimester of gestation using universal screening, according to the laboratory reference values, evaluated by enzyme chemo-luminescence immunoassay with the Beckman Coulter DXI 800 analyser (Table 1); and the prescription of thyroid hormone (group H03A of the Anatomical Therapeutic Chemical classification.

**Table 1:** Reference values of TSH and T4 in each trimester of pregnancy according to laboratory criteria

Trimester	TSH (nmol/L)	T4l (nmol/L)
First	0.50–3.70	6.70–16.30
Second	0.31–4.35	5.80–13.90
Third	0.41–5.18	6.10–15.80

Other variables studied were trimester of pregnancy, calculated from the date of the last menstrual period appearing in the medical record, the age of pregnant women body mass index (BMI), diabetes mellitus, arterial hypertension, dyslipidaemia, depression, pre-eclampsia and eclampsia, miscarriage, prematurity and caesarean delivery.

### Therapeutic adherence

Therapeutic adherence was analysed through the proportion of days covered (PDC) used by the “Pharmacy Quality Alliance” [16]. This proportion is defined as the percentage of days during which the patient receives thyroid hormone replacement therapy, with respect to the total period indicated by the guidelines.

Adherence during pregnancy was analysed from the date of the last menstruation period until the date of delivery, or in case of prematurity or miscarriage until the date of the end of the pregnancy.

Information on all reimbursable prescriptions was obtained from the pharmacy databases. Each prescription records the date of issue, the active ingredient prescribed, the number of units and the quantity of each drug. The indicated dose was obtained from the prescription made in the shared medical history.

Thus, as observed in other studies [17–20], we defined three levels of therapeutic adherence: high, for patients who took more than 80% of the drug prescribed; medium, for those who took between 50 and 80%; and low, for those who took < 50%.

### Analysis of data

A descriptive analysis was made. The numerical variables were indicated through mean and standard deviation, and the categorical variables by absolute and relative frequencies. Differences between groups were evaluated using the Student’s t test or the Chi-square test, depending on whether the variables were numerical or categorical, respectively. The association of the different variables with adherence was evaluated through a multivariate lineal model; the model was built using purposeful selection of variables based on clinical relevance, using the percentage of adherence as the response variable, and the rest of the variables as predictors. Regression coefficients and 95% confidence intervals of both were calculated.

### Ethical aspects

This study was approved by the ethics and clinical research committee “Institute for Primary Health Care Research Jordi Gol i Gurina (IDIAPJGol)” under the code 19/195-P. The study was conducted in accordance with the principles of the Declaration of Helsinki. Pseudonymized retrospective descriptive cross-sectional study according to Additional Provision 17.2.d LOPD-GDD for research purposes, without the need to obtain the consent of the data holders, there is a technical and functional separation between the research team and the performer pseudonymization, and that the data is only accessible to the research team, and technical measures have been taken to prevent such re-identification and access by third parties. through the CMBD database (“Conjunt Minim de Base de Dades”) the E-CAP computerized medical history database and the Catalan Health Service database.

## Results

### Epidemiological data

**Fig. 1:**
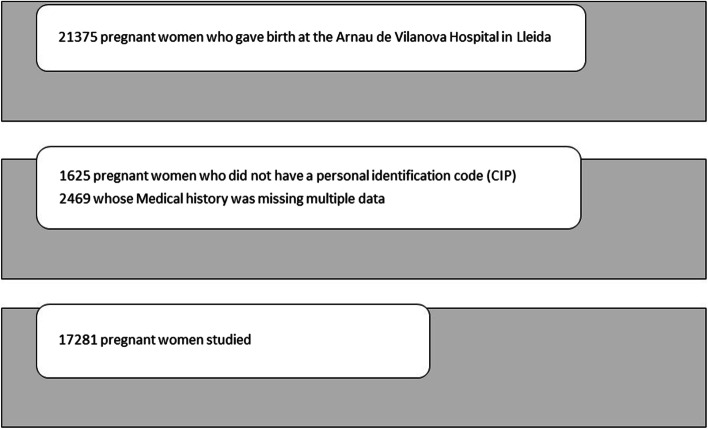
Sample of pregnant women studied

A total of 21,375 pregnant women who gave birth at the Arnau de Vilanova Hospital in Lleida between 2012 and 2018 (both included) were initially included in the study. Then, we excluded 1625 women who did not have a personal identification code (CIP), and 2469 women whose medical history was missing multiple data. The final sample comprehended 17,281 pregnant women (Fig. 1).

Of the total of 17,281 pregnant women, 1129 (6.52%) were diagnosed with hypothyroidism: 0.07% presented with clinical disease, and 6.45% with subclinical. The mean annual prevalence of pregnant women with hypothyroidism ranged from 5.67 to 7.05%. The mean age of these patients was 31.7 ± 5.7 years, in comparison to 30.6 ± 5.8 years of the rest of the study population. The BMI in patients at the beginning of pregnancy was 25 ± 5.24. 81.12% of these patients had one or two pregnancies during the study period. Of these, 20.8%, 36.3%, and 3.5% were classified as medium, high, or very high risk, respectively. Finally, 3.94% of the pregnant women with hypothyroidism had miscarriages, 6.64% preterm deliveries, 2.78% prolonged deliveries, and 16.74% underwent a caesarean section.

### Therapeutic adherence

Thyroid hormone treatment was prescribed in 50.3% of the patients diagnosed with hypothyroidism. Among them, the mean adherence score was 79.6 ± 22.2, and 54.1% of these presented high adherence. Specifically, during the years of the study, 40.4–64.7% of the treated patients showed high adherence.

The mean age was higher in treated patients with high adherence (32.8 years) than in those with low adherence (30.7 years). This difference was statistically significant. Regarding the other chronic diseases analysed, no statistically significant differences were observed in age between treated patients with high, medium, and low adherence. There were two miscarriages in the low adherence group and none in the medium and high adherence groups (Table 2).

**Table 2:** Adherence to the treatment of hypothyroidism and association with other variables studied during pregnancy

Adherence to the treatment	79.6% (ED 22.2)			N: 568
	High	Medium	Low	N: 568
Adherence to the treatment	306 (54.1%)	187 (32.9%)	74 (13.0%)	
Adherence per year				*p*: 0.830
2012	55 (64.7%)	17 (20.0%)	13 (15.3%)	
2013	60 (60.6%)	28 (28.3%)	11 (11.1%)	
2014	47 (58.8%)	24 (30.0%)	9 (11.2%)	
2015	39 (48.1%)	29 (35.8%)	13 (16.0%)	
2016	36 (40.4%)	44 (49.4%)	9 (10.1%)	
2017	36 (49.3%)	25 (34.2%)	12 (16.4%)	
2018	33 (55.0%)	20 (33.3%)	7 (11.7%)	
Age of pregnant women (years)	32.8 ± 4.77	32.1 ± 5.64	30.7 ± 6.24	*p*: 0.007
Body mass index	24.8 (5.26%)	25.4 (5.29%)	24.4 (5,62%)	*p*: 0.311
Association with other variables				
Diabetes Mellitus	46 (62.2%)	21 (28.4%)	7(9.4%)	*p*: 0.301
Arterial hypertension	10 (47.6%)	8 (38.1%)	3 (14.3%)	*p*: 0.774
Dyslipidaemia	6 (66.7%)	2 (22.2%)	1 (11.1%)	*p*: 0.890
Depression	12 (75.0%)	4 (25.0%)	0 (0.0%)	*p*: 0.195
Preeclampsia	4 (66.7%)	2 (33.3%)	0 (0.0%)	*p*: 1.000
Duration of the pregnancy				*p*: 0.129
Miscarriage	0 (0.0%)	0 (0.0%)	2 (100%)	
Preterm	22 (64.7%)	9 (26.5%)	3 (8.8%)	
Prolonged	7 (53.8%)	5 (38.5%)	1 (7.7%)	
At term	215 (52.5%)	143 (35.0%)	51 (12.5%)	
Caesarean delivery	56 (50.5%)	37 (33.3%)	18 (16.2%)	*p*: 0.493
Risk during pregnancy				*p*: 0.985
Really high	12 (54.5%)	7 (31.8%)	3 (13.6%)	
High	135 (56.5%)	73 (30.5%)	31 (13.0%)	
Medium	53 (55.2%)	29 (30.2%)	14 (14.6%)	
No risk	82 (51.6%)	54 (34.0%)	23 (14.5%)	

**Fig. 2:**
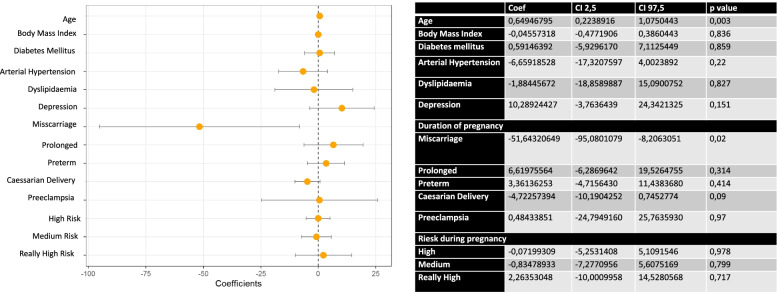
Multivariate analysis of adherence to treatment for hypothyroidism and its association with other variables

Figure 2 describes a multivariate analysis where statistically significant differences were observed with age: the older the patient, the greater the adherence to treatment. The rest of the related variables were not statistically significant.

### Value of TSH in relation to therapeutic adherence

Among patients showing high adherence to therapy, we observed poor TSH control in 66.8% during the first trimester, which dropped to 22% in the second, and finally to 8.3% in the third. On the contrary, in the low adherence group, poor control persists in the second and third trimesters. The differences between the two groups during the second trimester are statistically significant (Table 3).

**Table 3:** TSH values according to adherence to treatment

	High adherence	Medium adherence	Low adherence
1st trimester results TSH:			
High	191 (66.8%)	102 (64.6%)	41 (66.1%)
Low	5 (1.7%)	4 (2.5%)	0 (0.0%)
In range	90 (31.5%)	52 (32.9%)	21 (33.9%)
2nd trimester results TSH:			
High	58 (22.0%)	39 (25.2%)	13 (31.0%)
Low	5 (1.9%)	2 (1.3%)	0 (0.0%)
In range	201 (76.1%)	114 (73.5%)	29 (69.0%)
3rd trimester results TSH:			
High	18 (8.3%)	7 (5.2%)	6 (12.2%)
Low	10 (4.6%)	3 (2.2%)	2 (4.1%)
In range	190 (87.1%)	125 (92.6%)	41 (83.7%)

## Discussion

We examined a sample of 17,281 women, representing more than 92% of the pregnant women in the Lleida health region in the period analysed. The annual prevalence of hypothyroidism was 6.53% (0.07% clinical and 6.45% subclinical), with an oscillation between 5.67 and 7.05% in the different years. The mean age of these patients was 31.7 ± 5.7 years. Of the total of women in the sample, 3.3% received hormone replacement therapy. Among them, the mean adherence score was 79.6 ± 22.2. Those with high adherence had a higher mean age (32.8 years), in comparison to those with low adherence (30.7 years), being this difference statistically significant. Also, treated patients with low adherence had a higher abortion rate, even if with a wide confidence interval. Finally, high adherence to treatment was associated with better TSH control during pregnancy.

The prevalence of hypothyroidism in this study is higher in comparison to some other studies conducted in Spain, such as: the study by López Espinosa et. al. in the Valencia region in 2009, where the prevalence was 3.2% [4]; or the one by Diéguez et.al. in Asturias in 2016, where the prevalence was 5.5% (95% CI 4.6–6.3) [3]. This variability can be explained by differences in the population studied, as well as in our study, we analysed pregnant women, whereas the others evaluated the general population. In another study in pregnant women by Jaén Díaz, JL. performed in Toledo, the prevalence of hypothyroidism was higher than the one we found (9.5%, 95% CI 6–14.7) [21]. Moreover, in Europe, a study carried out in Belgium by Rodrigo Moreno-Reyes et al. showed a prevalence of hypothyroidism of 7.2% [22]. Finally, a meta-analysis in the Iranian population observed the highest prevalence of hypothyroidism during pregnancy (13.01%, 95% CI 9.15–18.17) [8], probably because of the lower degree of iodination in the population studied.

Various studies analysing adherence to treatment of multiple pathologies report different results. In a study in Denmark, data from the North Jutland prescription database were compared with the information provided by pregnant women through interviews carried out during the previous 120 days, and an adherence of 43% was described [14]. In agreement with it, another study on pregnant women at 36 weeks of gestation, which was carried out in Australia by means of a survey, obtained an adherence of 40.9% [15]. However, these data contrast with the ones obtained in studies that specifically analyse adherence to hypothyroid treatment in pregnant women. In this regard, in a cross-sectional multinational study carried out by Juch H. et. in 18 countries in 2016, it was reported that 39% of the treated patients had high adherence (95% CI, 32.7–45.7%); 44.1% medium (95% CI 12.5–22.5%); and 16.9% low (95% CI, 12.5–22.5%) [20]. In our study, we observe slightly higher degree of adherence: 54% high, 32.9% medium, and 13% low. In this case, the different percentages could be due to a methodological difference: we analysed the proportion of days covered (PDC), whereas the study by Juch H. et. al. used interviews [23].

In our study, the mean age of patients with high adherence was 32.8 ± 4.77 years. On the contrary, patients with low adherence were 30.7 ± 6.24 years old. Such difference is statistically significant, in agreement with the study by Juch H. et. al., that significantly associated young age with low adherence in pregnant women [23]. Moreover, in the study by Briesacher et. al., the analysis of adherence to treatment for various pathologies revealed that lower adherence was associated with younger age also in the general population [24].

There are few studies where TSH control is related to therapeutic adherence. In the study by Lage MJ et. al., the authors analysed 3448 pregnant women with hypothyroidism between 18 and 49 years. They observed that 52.61% of the women had a TSH value that was in the range established by the American Thyroid Association (ATA) guidelines [25]. In this regard, we observed differences according to the trimesters of pregnancy. Indeed, the prevalence of women showing TSH value in the range oscillated between 31.5% and 33.9% in the first trimester; 69% and 76.1% in the second trimester; and 83.7% and 92.6% in the third trimester, depending on the degree of therapeutic adherence. These results indicates that a sufficient control of the disease is obtained at the third trimester by patients showing different levels of adherence to treatment.

Finally, Lee SY et. al. concluded that both clinical and subclinical hypothyroidism are associated with abortions, prematurity, and low scores in the infant cognitive evaluation; and that the risk caused by the treatment necessary to maintain TSH in a specific reference range during pregnancy is minimal [26]. In our study, there was no association between low adherence and the appearance of complications during pregnancy; this may be due to the greater adherence and greater control of TSH during the last trimester. Also, Barišić T et. al. suggested that early detection and optimization of hypothyroidism treatment before and during the first trimester reduces the risk of adverse pregnancy outcomes [27]. In agreement with these data, in our study, greater adherence has been associated with greater TSH control; therefore, we consider important to involve the different professionals taking care of pregnant women, to improve therapeutic adherence. In poorly controlled patients, adherence should be assessed prior to adjusting the levothyroxine dose using the information provided by the patient [28].

### Difficulties and limitations of the study

Among the limitations of our study, we have to consider the loss of some cases during data collection. In particular, we missed pregnant women whose follow-up was carried out in centres that do not belong to the Social Security. However, it is estimated that they only represented around 2.2% of the total of pregnant women in the health region of Lleida [29]. Therefore, given the universal coverage of the Spanish National Health System, it is unlikely that this loss affected the results of our study.

Another limitation is that we could not to fully address the multifactorial origin of the adherence to treatment. Such multifactorial origin involve patient, family, beliefs, and psychosocial factors. All these variables should be considered in further studies.

## Conclusions

In this study, 6.53% of pregnant women were diagnosed with hypothyroidism and half of the women who followed the treatment showed good adherence. Patients with higher adherence were older and had better TSH control throughout pregnancy thant those with lower adherence. Overall, a high percentage of the treated pregnant women achieved well-controlled TSH levels in the third trimester.

Among the general population, adherence to treatment for chronic diseases is a complex issue because of the multiple factors involved. In the case of pregnancy, further factors may affect the mother’s decisions, such as the fear of additional adverse effects for herself and the newborn. Therefore, more evidence-based studies are necessary to provide information on the benefits of adherence to treatment.

## Supplementary Information

**Additional file 1. **Numberof births registered in the Lleida health region by years and number of birthsin the sample studied with the percentage they represent 

## Data Availability

De-identified survey data will be made available by emailing a request to: jvsiscart.lleida.ics@gencat.cat.
