# Supplementary material for: Adherence to treatment for hypothyroidism in pregnancy and relationship with thyrotropin control: a retrospective observational cohort study
Source: BMC Pregnancy Childbirth. 2022 Mar 1;22:168. doi: 10.1186/s12884-022-04483-8 (PMC8886742; doi:10.1186/s12884-022-04483-8)
Supplement: Supplementary file 1 — Additional file 1. Numberof births registered in the Lleida health region by years and number of birthsin the sample studied with the percentage they represent [file 12884_2022_4483_MOESM1_ESM.docx]

**Supplementary material**

| Supplementary table 1. Number of births registered in the Lleida health region by years and number of births in the sample studied with the percentage they represent | | | |
| --- | --- | --- | --- |
| Year | Deliveries from Idescat | Sample deliveries | Sample/Idescat |
| 2012 | 3788 | 3635 | 96% |
| 2013 | 3535 | 3370 | 95% |
| 2014 | 3592 | 3308 | 92% |
| 2015 | 3426 | 3162 | 92% |
| 2016 | 3283 | 3180 | 97% |
| 2017 | 3197 | 3034 | 95% |
| 2018 | 3029 | 3001 | 99% |
